# Longitudinal study based on a safety registry for malaria patients treated with artenimol–piperaquine in six European countries

**DOI:** 10.1186/s12936-021-03750-x

**Published:** 2021-05-08

**Authors:** Nicolas Vignier, Olivier Bouchaud, Andrea Angheben, Emmanuel Bottieau, Guido Calleri, Joaquín Salas-Coronas, Charlotte Martin, José Manuel Ramos, Matthieu Mechain, Christophe Rapp, Hans-Dieter Nothdurft, Maria Velasco, Azucena Bardají, Gerardo Rojo-Marcos, Leo G. Visser, Christoph Hatz, Zeno Bisoffi, Tomas Jelinek, Stephan Duparc, Yann Bourhis, Silva Tommasini, Maurizio Iannucelli, Antonella Bacchieri, Giovan Giuseppe Mattera, Emilio Merlo Pich, Ronald H. Behrens

**Affiliations:** 1 Department of Infectious and Tropical Diseases, and Laboratoire Éducations et Pratiques de Santé (LEPS EA 3412), Sorbonne Paris Nord University, Avicenne Hospital, Assistance Publique-Hôpitaux de Paris (AP-HP), Bobigny, France; 2 Centre D’Investigation Clinique Antilles-Guyane, Inserm 1424, Centre Hospitalier de Cayenne, Cayenne, France; 3 INSERM, Sorbonne Université, Institut Pierre Louis D’Épidémiologie et de Santé Publique IPLESP, Paris, France; 4 TropNet, A European Network for Tropical and Travel Medicine, Verona, Italy; 5 Department of Infectious-Tropical Diseases and Microbiology, IRCCS Sacro Cuore Don Calabria Hospital, Negrar, Italy; 6 Institute of Tropical Medicine, Antwerp, Belgium; 7 Azienda Sanitaria Locale “Cità Di Torino”, Torino, Italy; 8 Tropical Medicine Unit, Hospital de Poniente, El Ejido, Almería Spain; 9 Travel Clinic, CHU St Pierre, Brussels, Belgium; 10 Consulta de Enfermedades Importadas Y Parasitología Clínica, Unidad de Enfermedades Infecciosas, Hospital General Universitario Alicante, Alicante, Spain; 11 Hôpital St André, Bordeaux, France; 12 Hôpital D’Instruction Des Armées Begin, Saint Mandé, France; 13 Klinikum Der LMU München, Munich, Germany; 14 Hospital Universitario Fundación Alcorcón, Madrid, Spain; 15 ISGlobal, Hospital Clínic, Universitat de Barcelona, Barcelona, Spain; 16 Centro de Investigação Em Saúde de Manhiça, Maputo, Mozambique; 17 Consorcio de Investigación Biomédica en Red de Epidemiología Y Salud Pública (CIBERESP), Madrid, Spain; 18 Hospital Universitario Príncipe de Asturias, Alcalà de Henares, Madrid, Spain; 19 Department of Infectious Diseases, Leiden University Medical Centre, Leiden, Netherlands; 20 Swiss Tropical and Public Health Institute, Basel, Switzerland; 21 University of Basel, Basel, Switzerland; 22 Berliner Centrum Fürr Reise- Und Tropenmedizin, Berlin, Germany; 23 Medicines for Malaria Venture, Geneva, Switzerland; 24 Mapi Group, Real World Evidence, Lyon, France; 25 Research & Development, Alfasigma S.P.A, Bologna, Italy; 26 Clinical Research Dept, Faculty of Infectious & Tropical Diseases, London School of Hygiene and Tropical Medicine, London, UK

**Keywords:** Imported malaria, Artenimol, Piperaquine, Eurartesim, QTc prolongation, Safety, Adverse events, Artemisinin, *Plasmodium falciparum*

## Abstract

**Background:**

European travellers to endemic countries are at risk of malaria and may be affected by a different range of co-morbidities than natives of endemic regions. The safety profile, especially cardiac issues, of artenimol (previously dihydroartemisinin)–piperaquine (APQ) Eurartesim^®^ during treatment of uncomplicated imported falciparum malaria is not adequately described due to the lack of longitudinal studies in this population. The present study was conducted to partially fill this gap.

**Methods:**

Participants were recruited through Health Care Provider’s safety registry in 15 centres across 6 European countries in the period 2013–2016. Adverse events (AE) were collected, with a special focus on cardiovascular safety by including electrocardiogram QT intervals evaluated after correction with either Bazett’s (QTcB) or Fridericia’s (QTcF) methods, at baseline and after treatment. QTcB and/or QTcF prolongation were defined by a value > 450 ms for males and children and > 470 ms for females.

**Results:**

Among 294 participants, 30.3% were women, 13.7% of Caucasian origin, 13.5% were current smoker, 13.6% current alcohol consumer and 42.2% declared at least one illness history. The mean (SD) age and body mass index were 39.8 years old (13.2) and 25.9 kg/m^2^ (4.7). Among them, 75 reported a total of 129 AE (27 serious), 46 being suspected to be related to APQ (11 serious) and mostly labelled as due to haematological, gastrointestinal, or infection. Women and Non-African participants had significantly (p < 0.05) more AEs. Among AEs, 21 were due to cardiotoxicity (7.1%), mostly QT prolongation, while 6 were due to neurotoxicity (2.0%), mostly dizziness. Using QTcF correction, QT prolongation was observed in 17/143 participants (11.9%), only 2 of them reporting QTcF > 500 ms (milliseconds) but no clinical symptoms. Using QTcB correction increases of > 60 ms were present in 9 participants (6.3%). A trend towards increased prolongation was observed in those over 65 years of age but only a few subjects were in this group. No new safety signal was reported. The overall efficacy rate was 255/257 (99.2%).

**Conclusions:**

APQ appears as an effective and well-tolerated drug for treatment of malaria in patients recruited in European countries. AEs and QT prolongation were in the range of those obtained in larger cohorts from endemic countries.

*Trial registration* This study has been registered in EU Post-Authorization Studies Register as EUPAS6942

**Supplementary Information:**

The online version contains supplementary material available at 10.1186/s12936-021-03750-x.

## Background

Imported malaria into non-endemic regions, particularly Europe and North America is an ongoing problem and most of the cases are in travellers visiting family and relatives [1]. Treatment regimens which are efficacious, well-tolerated and with a simple administration schedule, to improve treatment out of hospital settings, are limited. The combination of artenimol 40 mg (previously dihydroartemisinin) and piperaquine tetraphosphate 320 mg (APQ), are marketed as Eurartesim^®^ by Alfasigma (Italy) worldwide. It is an effective artemisinin-based combination therapy which involves the simultaneous use of two blood schizontocidal compounds with independent modes of action, meeting all these needs [2, 3].

The most common side-effects observed with APQ use in uncomplicated malaria patients (1–10 patients in 100) are anaemia, headache, corrected QT segment (QTc) prolongation and tachycardia [4].

Preclinical studies with artemisinin-related products in rats and dogs showed electrocardiographic (ECG) effects, in particular prolongation of QTc [5–7]. Conversely, exposure to piperaquine was not associated with relevant ECG abnormalities [8]. Artesunate has not been associated with QT prolongation in clinical use [9]. Malaria illness itself may also affect the heart rate and QT interval [10]. The QT prolongation seen with APQ is a piperaquine effect which has been demonstrated in pre-clinical and clinical studies [11, 12]. QT prolongation is a sensitive, but not specific indicator of increased risk of the polymorphic ventricular tachycardia, torsades de pointes which can degenerate into cardiac arrest rhythms and cause sudden cardiac death. Therefore, the QTc prolongation risk was monitored during the clinical development of APQ, specifically in the pivotal clinical trials DM040010 and DM040011, where APQ was compared to loose combination of artesunate + mefloquine and fixed dose combination of artemether and lumefantrine regimen, respectively [13, 14]. In these studies, ECGs were performed on days 0 and 2 (last treatment day) and 7 after starting treatment. QTc interval was analysed using standard metrics according to ICH E14 guideline using the Bazett’s method correction (QTcB) [15]. At baseline a certain number of QTc prolongation were observed and associated with the malaria infection. By day 2, a higher proportion of patients with prolonged QTc values were observed in the APQ group vs. comparators. However, only 7 subjects out of the 1805 patients included in the two trials had a QTc > 500 ms (milliseconds) (0.38%). These changes were reversible, where by day 7, these differences disappeared. None of the prolongated QTc was associated with any clinically relevant arrhythmic event. Other studies showed less QTc prolongations after APQ treatment in fasting conditions [21.0 ms (15.7–26.4) vs 46.0 ms (39.6–52.3) with high-fat/high caloric breakfast] [4, 16].

Following these studies the European marketing authorization for APQ was obtained for the treatment of uncomplicated *Plasmodium falciparum* malaria, including a warning not to take food within 3 h of ingesting APQ. The drug should not be prescribed to patients with risk factors for QTc prolongation (i.e. family history of sudden death or of congenital prolongation of the QTc interval, known congenital prolongation of the QTc-interval, any clinical condition known to prolong the QTc interval). These include a history of symptomatic cardiac arrhythmias, clinically relevant bradycardia, any predisposing cardiac conditions for arrhythmia, electrolyte disturbances, and recent or on-going treatment known to prolong the QTc interval [4].

While data from its use in malaria-endemic areas supports a very low risk of sudden death after APQ, which is not higher than the population baseline risk, there are few data available on APQ used for the treatment of uncomplicated malaria in returning travellers in Europe [17].

In order to assess the actual safety profile and the potential for cardiotoxicity and QTc prolongation following APQ exposure in these patients, a European post-authorization safety registry was proposed with the goal to monitor all incoming patients with imported malaria. The present work is summarizing the results obtained in a study performed in patients from this registry.

## Methods

### Study design and participants

This study was an observational, registry-based, longitudinal, multicentric study assessing the safety profile of uncomplicated malaria patients receiving APQ treatment. Participants to the study were selected among patients included in a Health Care Provider (HCP) Safety Registry organized to monitor the outcome and safety of APQ treatment, consisting of 15 clinical centres across 6 European countries (Belgium, France, Germany, Italy, Spain and the UK) authorized for the use of APQ. Standard APQ treatment was given at the manufacturer recommended dosage of 320 mg/40 mg coated-film tablets of either three tablets for participants weighting < 75 kg or four tablets in participants weighted ≥ 75 kg, administered three times at 24 h interval for 3 consecutive days.

Participants were selected based on the following inclusion criteria: clinical and parasitological (microscopy or PCR) diagnosis of uncomplicated *P. falciparum* malaria that met the summary of product characteristics indications, any gender and age, and signed informed consent and evidence of receiving the APQ treatment on the day of enrolment [4]. Each HCPs were encouraged to include as many patients meeting the eligibility criteria. Paediatric patients (5 months to 18 years of age) could also be enrolled for inclusion in the registry with the child’s parent or legal guardian informed consent. To ensure sufficient power to conduct the analysis three hundred patients were considered an appropriate sample to reflect the drugs adverse events profile.

Safety data were collected during the normal course of patient care by HCPs preferably during a 3-day hospitalization or, when hospitalization was refused or not possible, on an ambulatory basis. An ECG recording was recommended at baseline after patient enrolment (visit 1 defined as baseline day before or at the time of the first APQ administration) and after the last APQ dose (visit 2 = at the ‘final treatment day’, defined as the day of last APQ administration, applying a flexibility time-window of maximum 2 days in case of missing ECG assessment at the day of last APQ administration). For the purposes of the analysis, the subpopulation having an ECG on the ‘strict final treatment day’, defined as the day of last APQ administration, was also individualized and named ‘Strict QTc population’. A follow-up visit (visit 3) was planned between 3 and 5 weeks after hospital discharge or after the out-patient treatment. If the visit was not feasible, patients were contacted by an independent study monitor for follow-up between day 15 and day 45. Any AE and concomitant medication were recorded.

### Safety outcome measurements

The primary objective was to study the safety profile associated to APQ treatments. The frequency and severity of all adverse events (AE) was recorded, with a particular attention to the main AE of Special Interest (AESI) for AQP treatment, i.e., cardiotoxicity (syncope, palpitations, chest pain, torsade de pointes, ventricular fibrillation, ventricular tachycardia and sustained arrhythmias), neurotoxicity (abnormal behaviour, convulsion, dizziness, febrile convulsion, hallucination, stroke, paresthesia or tinnitus), and phototoxicity (dermatitis or rash). Investigators were trained in AE/AESI reporting and had to specify if AEs were suspected to be related to APQ, and report the action taken and the outcome. Differences between pre-treatment baseline values and post-treatment values were measured for blood chemistry markers, such as serum alanine amino-transferase (ALAT), aspartate amino-transferase (ASAT) and creatinine, and for ECG recording parameters, specifically the QTc intervals. Measurements of blood chemistry markers such as serum ALAT, ASAT and creatinine were carried out in at each centre using standard methodologies.

Measurements of ECG-based QTc interval (ms) were evaluated after correcting for the heart rate with Bazett’s (QTcB) and Fridericia’s formulae (QTcF) [15]. QTc prolongation were defined by a value > 450 ms for males and children and > 470 ms for females [18]. Borderline QTc was defined 430–450 ms for males and 430–470 for females. The QTc increase from baseline and the proportion of increase > 60 ms were also studied. Other factors were also recorded using a questionnaire for multivariate analysis: age, gender, ethnicity, lifestyle (smoking status, alcohol consumption), co-morbidities and co-medications. Information about the time between intake of food and APQ dosing was also collected. The cure rate was defined as negative parasitaemia at Visit 3.

### Statistical analyses

Three populations were analysed: (1) the general ‘safety population’ that include all subjects receiving at least one dose of APQ; (2) the ‘QTcF/QTcB population’ defined as all patients having at least two ECG, one at baseline-visit 1 and one at the ‘final treatment day’, and (3) the ‘Strict QTcF/QTcB population’ with the second ECG at the ‘strict final treatment day’.

Data were reported and tabled as mean + SD. Regarding the ECG measurements, differences in value of QTcF and QTcB were compared using ANOVA (p < 0.05).

Change in the levels of ALAT, ASAT and creatinine were analysed with a non-parametric test of Wilcoxon’s. Multivariate analysis was conducted with ANOVA including all covariates with a p < 0.20 and using a Backward selection process with a p < 0.05. The variables considered are the characteristics of the patients likely to or that could be associated with a variability of the QT space (gender, age, ethnicity, smoking status, alcohol consumption, time between APQ administration and last meal, co-medications known to prolong QT, liver abnormalities and renal abnormalities at baseline).

### Sample size

A sample size of 300 patients was selected to ensure a large enough sample to capture the association between safety parameters (in particular QTc prolongation) and pre-specified factors, and to have a 90% probability of observing at least one cardiac AESI assuming an overall true incidence of these events of 0.008.

### Ethics approval and consents to participate

The study followed a centralized regulatory submission at the European Medicines Agency (Approval 2013, prot. n° 3381). Ethical approval was received also by Hospital Ethics Committee in all the Countries participating in the study. Patients were enrolled after Informed consent signature.

## Results

**Fig. 1:**
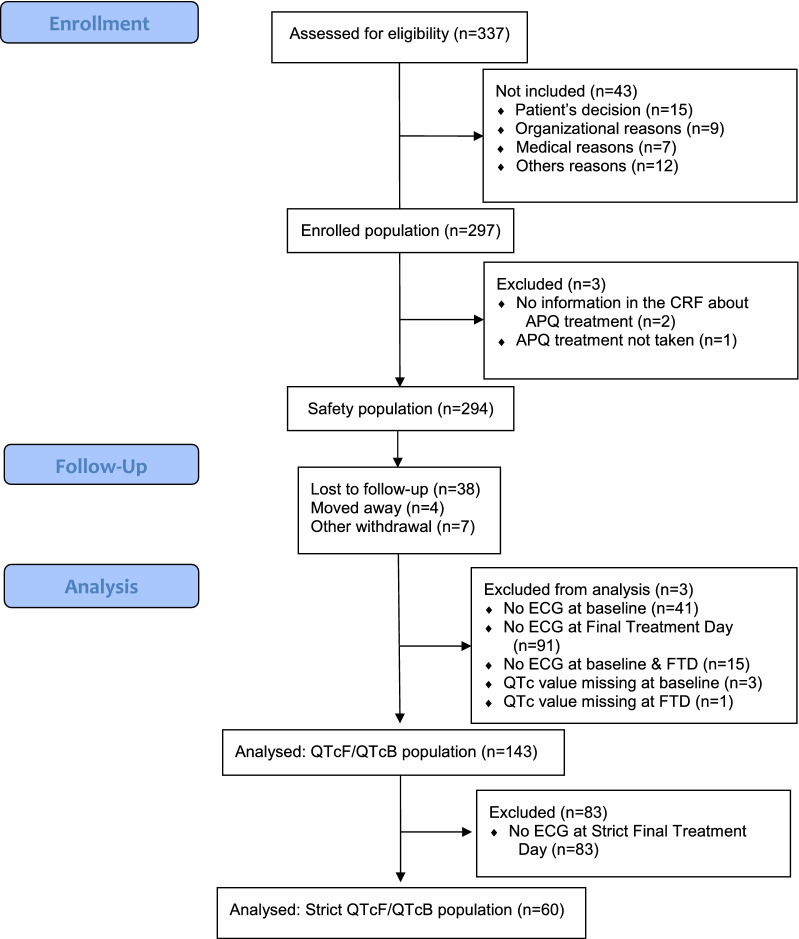
Flow diagram—safety registry of malaria patients treated with artenimol–piperaquine

From May 2013 to August 2016, a total of 297 patients were included by 15 active centres (among 29 contacted and 23 agreed to participate): two centres in Belgium (n = 59 patients included), three in France (n = 93), two in Germany (n = 12), two in Italy (n = 77), five in Spain (n = 52) and one in the UK (n = 4). Most of active centres were located at university hospitals (80.0%) in infectious and tropical diseases departments (66.6%). Additionally, 43 patients meeting the selection criteria were not included in the study because patient’s decision (34.3%), organizational reasons (20.0%) and medical reasons (17.1%) (Fig. 1).

Of these 297 patients, 294 patients received at least one dose of APQ and were considered in the analysis of the safety population registry (two patients were excluded because no information regarding APQ treatment was included in the CRF and one patient because he never received the drug). A total of 49 patients prematurely discontinued the study (38 lost to follow up, four moved away and seven for other reasons), most often after the final treatment day (Fig. 1). They were considered in the analysis when information was available.

### Characteristics of participants

Women accounted for 30.3% of the participants (Table 1). The majority of patients were of African origin (84.6%) or Caucasian (13.7%). The mean age of patients was 39.8 years (SD: 13.2) with 3.4% 66–74 years old. The mean body weight of adult was 77.2 kg (range 46–121) with 26 patients weighed at least 100 kg.

**Table 1:** Characteristics of participants—safety registry of malaria patients treated with artenimol-piperaquine

	Safety population	QTcF population	Strict QTcF population
	N = 294	N = 143	N = 60
Age (years)			
Mean (SD)	39.8 (13.2)	37.5 (12.4)	37.7 (12.1)
Range	1.5; 74.0	12.0; 72.0	12.0; 72.0
Age category			
≤ 12 years old	5 (1.7%)	1 (0.7%)	1 (1.7%)
13–17 years old	5 (1.7%)	5 (3.5%)	2 (3.3%)
≥ 18 years old	284 (96.6%)	137 (95.8%)	57 (95.0%)
Gender			
Male	205 (69.7%)	98 (68.5%)	42 (70.0%)
Female	89 (30.3%)	45 (31.5%)	18 (30.0%)
Ethnicity			
Missing	1		
Oriental	1 (0.3%)		
African	248 (84.6%)	118 (82.5%)	51 (85.0%)
Caucasian	40 (13.7%)	24 (16.8%)	8 (13.3%)
Hispanic	1 (0.3%)	1 (0.7%)	1 (1.7%)
Other	3 (1.0%)		
Body Mass Index at visit 1 (kg/m^2^)			
Mean (SD)	25.9 (4.7)	25.6 (4.6)	26.1 (5.2)
Range	15.7; 42.2	17.1; 13.9	18.2; 37.8
Smoking status			
Missing	12	5	3
Never smoked	205 (72.7%)	101 (73.2%)	40 (70.2%)
Previous smoker	39 (13.8%)	16 (11.6%)	9 (15.8%)
Current smoker	38 (13.5%)	21 (15.2%)	8 (14.0%)
Alcohol consumption			
Missing	14	5	3
Never/on special occasions (less than once a week)	237 (84.6%)	115 (83.3%)	45 (78.9%)
Ex-consumer	5 (1.8%)	3 (2.2%)	2 (3.5%)
Current consumer	38 (13.6%)	20 (14.5%)	10 (17.5%)
At least one illness history			
Yes (cf Additional file for details)	124 (42.2%)	55 (38.5%)	26 (43.3%)
No	170 (57.8%)	88 (61.5%)	34 (56.7%)
Clinical symptoms at enrolment			
Yes	278 (94.6%)	138 (96.5%)	56 (93.3%)
No	16 (5.4%)	5 (3.5%)	4 (6.7%)
Symptoms			
Fever	261 (88.8%)	128 (89.5%)	50 (83.3%)
Headache	195 (66.3%)	99 (69.2%)	41 (68.3%)
Shivering	146 (49.7%)	78 (54.5%)	33 (55.0%)
Arthralgia	137 (46.6%)	72 (50.3%)	27 (45.0%)
Sweating	110 (37.4%)	55 (38.5%)	21 (35.0%)
Vomiting	107 (36.4%)	49 (34.3%)	15 (25.0%)
Anemia	26 (8.8%)	11 (7.7%)	7 (11.7%)
Jaundice	14 (4.8%)	6 (4.2%)	4 (6.7%)
Splenomegaly	14 (4.8%)	8 (5.6%)	1 (1.7%)
Hepatomegaly	3 (1.0%)	3 (2.1%)	3 (5.0%)
Retinal damage	0	0	0
Coma	1 (0.3%)	0	0
Other symptom	103 (35.0%)	51 (35.7%)	23 (38.3%)

QTcF population: patients with corrected QT interval calculated using Fridericia’s formulae, SD: Standard deviation Strict Strict QTcF/B population: patients with corrected a QT interval calculated at the strict final treatment day

Parasitological diagnosis was predominantly by thin film microscopy for *P. falciparum* (n-273) by PCR (n = 6), thick smear (n = 4), *P. falciparum* antigen (n = 1), and positive but with unknown method for others (n = 4). Median *P. falciparum* counts were 25.0 × 10^3^/µL (IQR: 3.5–80.0) (n = 273). Co-infection with other species were identified in 6 patients (*Plasmodium vivax,* n = 1; *Plasmodium ovale,* n = 2*; Plasmodium malariae,* n = 3). The most frequent symptoms of malaria were fever and headache. Haemoglobin was below the lower normal range for 38.2% of participants at baseline (Additional file 2). Liver and renal abnormalities were reported for 21.8% and 5.0% of participants, respectively.

Overall, 42.2% had a history of at least one comorbidity, of which 37.9% had hypertension, 10.5% diabetes, 14.5% HIV infection and 6.5% cardiac disorders (including 2 atrial fibrillations, 1 arrhythmia and 1 cardiac hypertrophy). Details of comorbidities can be viewed in (Additional file 1).

Among the 254/294 (86.4%) of patients who took concomitant medications at baseline, the most frequent were analgesics (77.2%), drugs for functional gastrointestinal disorders (21.3%), and antibiotics (21.3%). In total, between the month prior and the last day of APQ, 27.6% of participants had taken treatments known to prolong the QTc interval.

APQ was initiated on median the day of malaria diagnosis (IQR: 0–1) and 97.3% of patients received APQ for 3 days. APQ dosages were generally consistent with patient weight (minor inconsistencies, observed in 22 patients (7.9%), mainly 4 tablets per day for patient < 75 kg). The 24 patients but one weighing 100 kg or more received adequate dosage (12 pills). The APQ administration more than 3 h from food intake was respected in 182 of the 267 patients (68.2%) in whom information was available.

### Parasitological outcome

The *P. falciparum* overall efficacy rate was 99.2% with 255/257 having eliminated the parasitaemia at follow-up. Two patients were considered to have developed a recurrence of malaria patients as they were found to have a positive parasitaemia at visit 3.

### Safety parameters in safety population

No woman became pregnant after APQ administration. No substantial changes in hemoglobin and hematocrit were observed. Neutrophil granulocyte, platelet counts, liver parameters and C-reactive protein tended to improve during the course of the study. A total of 129 AEs were experienced by 75/294 patients (25.5%), of which 9 severe AE. In addition, 27 were serious AE were reported in the study (25 led to hospitalization/prolonged hospitalization, 11 to the prescription of corrective medications, 4 to APQ permanent discontinuation and 2 to another action) (Additional file 3). Regarding the relationship with APQ, 46 were suspected to be related (Table 2), 11 of them were defined as serious AE (Table 3) and mostly labelled as due to haematological (anaemia, haemolysis), gastrointestinal (vomiting, acute hepatitis), or infection (malaria, encephalitis brain stem) (Additional file 3).

**Table 2:** Overview of adverse events (AE) other than adverse event of special interest (AESI) suspected related to APQ by System Organ Class and Preferred Term

System Organ Class (MedDRA) Preferred Term (MedDRA)	Safety population	
	Patients^a^ N = 294	Events N = 46
Blood and lymphatic system disorders	4 (1.4%)	4
Haemolysis	2 (0.7%)	2
Anaemia	1 (0.3%)	1
Haemolytic anaemia	1 (0.3%)	1
Gastrointestinal disorders	16 (5.4%)	20
Vomiting	9 (3.1%)	9
Diarrhoea	4 (1.4%)	4
Abdominal pain	3 (1.0%)	3
Nausea	2 (0.7%)	2
Gastrointestinal pain	1 (0.3%)	2
General disorders and administration site conditions	2 (0.7%)	2
Asthenia	1 (0.3%)	1
Malaise	1 (0.3%)	1
Hepatobiliary disorders	2 (0.7%)	2
Hepatitis	1 (0.3%)	1
Hepatitis acute	1 (0.3%)	1
Infections and infestations	3 (1.0%)	3
Encephalitis brain stem	1 (0.3%)	1
Malaria	1 (0.3%)	1
Plasmodium falciparum infection	1 (0.3%)	1
Musculoskeletal and connective tissue disorders	4 (1.4%)	4
Back pain	2 (0.7%)	2
Myalgia	1 (0.3%)	1
Rhabdomyolysis	1 (0.3%)	1
Nervous system disorders	4 (1.4%)	7
Headache	3 (1.0%)	6
Insomnia	1 (0.3%)	1
Respiratory, thoracic and mediastinal disorders	2 (0.7%)	2
Dyspnoea	2 (0.7%)	2
Skin and subcutaneous tissue disorders	2 (0.7%)	2
Dry skin	1 (0.3%)	1
Pruritus	1 (0.3%)	1

An event is considered as suspected related to APQ if the relationship with APQ is 'related', 'suspected', 'unassessable' or 'missing' as reported by the investigator in the 'Adverse event' section of the CRF

*AE* Adverse event, *SAE* serious adverse event, *AESI* adverse event of special interest, *APQ* artenimol–piperaquine, *MedDRA* AEs are coded using MedDRA dictionary version 16.0

^a^If one patient presented an event several times (same preferred term), s/he is counted once for that term. The same rule applies for results by System Organ Class

**Table 3:** Overview of serious adverse events (AE) other than adverse event of special interest (AESI) suspected related to APQ by System Organ Class and Preferred Term

System Organ Class (MedDRA) Preferred Term (MedDRA)	Safety population	
	Patients^a^ N = 294	Events N = 11
Blood and lymphatic system disorders	3 (1.0%)	3
Haemolysis	2 (0.7%)	2
Anaemia	1 (0.3%)	1
Gastrointestinal disorders	2 (0.7%)	3
Vomiting	2 (0.7%)	2
Nausea	1 (0.3%)	1
General disorders and administration site conditions	1 (0.3%)	1
Malaise	1 (0.3%)	1
Hepatobiliary disorders	2 (0.7%)	2
Hepatitis	1 (0.3%)	1
Hepatitis acute	1 (0.3%)	1
Infections and infestations	2 (0.7%)	2
Encephalitis brain stem	1 (0.3%)	1
Plasmodium falciparum infection	1 (0.3%)	1

An event is considered as suspected related to APQ if the relationship with APQ is 'related', 'suspected', 'unassessable' or 'missing' as reported by the investigator in the 'Adverse event' section of the CRF

*AE* Adverse event, *SAE* serious adverse event, *AESI* adverse event of special interest, *APQ* artenimol–piperaquine, *MedDRA* AEs are coded using MedDRA dictionary version 16.0

^a^If one patient presented an event several times (same preferred term), s/he is counted once for that term. The same rule applies for results by System Organ Class

Of the AE of special interest (AESIs), 28 occurred in 27 patients (9.2%). Among those reported, 21 cases (7.1%) were relation to cardiac abnormality (prolonged QTc, n = 19, ventricular tachycardia, n = 1, palpitation, n = 1). The clinical details on the subject with ventricular tachycardia; a 36-year-old black male with a previous diagnosis of athlete cardiomyopathy and known ECG abnormalities. The subject had never smoked and consumed alcohol less than once a week. This patient, at presentation, described abdominal pain, influenza like symptoms, fever, fatigue, arthralgia, myalgia, vomiting and headache. At baseline *P. falciparum* count was 0.5 10e3/μL. He presented with pre-treatment liver enzyme abnormalities (Aspartate amino-transferase 79 mIU/mL) and normal renal function creatinine 12.7 mg/L. He had taken paracetamol 500 mg/ascorbic acid 200 mg/pheniramine maleate 25 mg prior to presentation. Following the diagnosis of malaria, he received the first dose of 3 tablets 320/40 mg of APQ adjusted to his weight of 65 kg (BMI 20.7 kg/m^2^). The time of his last meal to dosing was unknown. His fever and nausea were treated with metoclopramide 10 mg IV and paracetamol 1000 mg IV on the first day. His QTcF/QTcB were within the normal range at baseline and at his 2nd ECG on day 2, after APQ, the QTcF/QTcB changes were 5 and 9 ms, respectively. The ECG PR interval and QRS were in normal range at baseline and after APQ. Because of his vomiting, he was switched to atovaquone/proguanil at day 2 and for the subsequent 3 days (incomplete course of APQ). Five days after the last dose of APQ and 1 day after last dose of atovaquone/proguanil, he presented with an episode of ventricular tachycardia which reverted spontaneously. He was followed 48 days without a similar event. The ventricular tachycardia was judged to be related to his cardiomyopathy and not to the APQ treatment.

Others cardiac AESIs were of mild intensity and of which 19 were considered related to APQ and 3 led to drug discontinuation. In addition, 6 cases (2.0%) of neurotoxicity (dizziness, n = 4, hallucination, n = 1, paraesthesia, n = 1) were reported of which 4 cases were mild and 2 were of moderate intensity; 4 of them were considered APQ-related. Finally, 1 case (0.3%) of phototoxicity (rash of mild intensity) was raised, recovered after 3 weeks and was not considered drug related. Due to their rare occurrence, it was not possible to perform a factor analysis associated with AESIs.

According to multivariate analysis, non-African patients and females were more likely to experience AEs (including AESIs) than African patients and males (p < 0.05), respectively (Additional file 3). Moreover, in univariate analysis, the percentage of patients who experienced at least one AE (including AESIs) suspected to be related to APQ was lower (p < 0.05) in African patients and in patients without renal abnormalities (Additional file 4).

### QTc prolongation

At baseline, among the 234 out of 237 patients with available ECG information, QTcF/QTcB values were normal for the majority of patients. Prolonged and borderline QTcF were observed in four (1.7%) and 10 (4.3%) patients, respectively. These figures were 13 (5.5%) and 27 (11.4%) for QTcB. Only one patient (male, 33 years) had a QTc value > 500 ms at baseline (QTcB = 557 ms and QTcF = 478 ms), before APQ administration, but was lost to follow up without another ECG.

The ‘QTcF/QTcB population’ was of 143 out of 234 participants, whereas the ‘strict QTcF/QTcB population” was of 60 out of 143.

ECG changes are detailed in Table 4. In the ‘QTcF/QTcB population’, a QTc prolongation at final treatment day was observed for 5 patients/143 (3.4%) for QTcF and 9/143 (6.3%) for QTcB. In the ‘strict QTcF/QTcB population’, a QTc prolongation at the strict final treatment day was observed for 4 patients/60 (6.7%) for QTcF and 5/60 (8.3%) for QTcB. Increase > 60 ms in value from baseline to visit 2 was reported for 9 and 2 patients for QTcF and QTcB (Table 4). Over the follow-up period, QTc values > 500 ms were reported in two patients: one on the second day (QTcB = 510 ms and QTcF = 501 ms) and one on the third day (QTcB = 531 ms and QTcF = 425 ms). The first patient was an African male (66 years) with a QTcB/QTcF already prolonged at baseline (557 /478 ms) and a QTc assessment performed 4 h after first APQ administration with food intake less than 3 h from APQ and concomitant terbutaline intake. QTcF/QTcB returned to normal on the third day. The second patient was a 19-year-old African man with a QTcB at 413 ms at baseline before APQ and no other risk factors The QT anomalies resolved within 24 h and had no clinical consequences.

**Table 4:** ECG after treatment administration: QTcB and QTcF (continuous parameter in ms)—Safety, QTcF/QTcB and strict QTcF/QTcB population

	Safety population		QTcF/QTcB population		Strict QTcF/QTcB population	
	N = 294		N = 143		N = 60	
	QTcF	QTcB	QTcF	QTcB	QTcF	QTcB
Baseline						
N	234	237	143	143	60	60
Missing	60	57	0	0	0	0
Mean (SD)	387.8 (29.6)	410.8 (32.3)	385.3 (28.6)	408.5 (29.8	386.8 (32.8)	407.7 (33.4)
Range	290; 478	290; 557	299; 470	309; 486	299; 470	309; 480
Final treatment day						
N	186	186	143	143		
Missing	108	108	0	0		
Mean (SD)	405.9 (28.8)	413.9 (31.8)	404.2 (29.1)	411.8 (32.3)		
Range	299; 494	289; 500	299; 494	289; 500		
Strict final treatment day						
N	71	71	60	60	60	60
Missing	223	223	83	83	0	0
Mean (SD)	406.7 (36.2)	411.7 (38.8)	404.3 (35.3)	410.2 (38.4)	404.3 (35.3)	410.2 (38.4)
Range	299; 494	289; 500	299; 494	289; 500	299; 494	289; 500
Absolute change from baseline to final treatment day						
N	143	146	143	143		
Missing	151	148	0	0		
Mean (SD)	18.9 (24.7)	4.0 (26.7)	18.9 (24.7)	3.3 (25.0)		
Range	− 47; 81	− 68; 116	− 47; 81	− 68; 80		
Absolute change from baseline to strict final treatment day						
N	60	62	60	60	60	60
Missing	234	232	83	83	0	0
Mean (SD)	17.5 (18.9)	4.7 (26.7)	17.5 (18.9)	2.6 (22.8)	17.5 (18.9)	2.6 (22.8)
Range	− 23; 69	− 46; 116	− 23; 69	− 46; 80	− 23; 69	− 46; 80
Change in QTcF/QTcB in classes from baseline to final treatment day						
Missing	151	148	0	0		
Strict decrease or no change	28 (19.6%)	65 (44.5%)	28 (19.6%)	64 (44.8%)		
Increase < 30 ms	70 (49.0%)	59 (40.4%)	70 (49.0%)	58 (40.6%)		
Increase between 30 and 60 ms	36 (25.2%)	20 (13.7%)	36 (25.2%)	20 (14.0%)		
Increase > 60 ms	9 (6.3%)	2 (1.4%)	9 (6.3%)	1 (0.7%)		
Change in QTcF/QTcB in classes from baseline to strict final treatment day						
Missing	234	232	83	83	0	0
Strict decrease or no change	10 (16.7%)	30 (48.4%)	10 (16.7%)	30 (50.0%)	10 (16.7%)	30 (50.0%)
Increase < 30 ms	36 (60.0%)	24 (38.7%)	36 (60.0%)	23 (38.3%)	36 (60.0%)	23 (38.3%)
Increase between 30 and 60 ms	12 (20.0%)	6 (9.7%)	12 (20.0%)	6 (10.0%)	12 (20.0%)	6 (10.0%)
Increase > 60 ms	2 (3.3%)	2 (3.2%)	2 (3.3%)	1 (1.7%)	2 (3.3%)	1 (1.7%)

QTcF/B population: patients with corrected QT interval calculated using Fridericia’s formulae/Bazett’s formulae; Strict QTcF/B population: patients with corrected a QT interval calculated at the strict final treatment day

The mean change in QTcF and QTcB from baseline to the strict final treatment day (n = 60) was + 17.5 and + 2.6 ms, respectively. Multivariate assessment of the factors associated to changes in the eligible population is shown in Table 5. Patients who never smoked had a statistically significantly (p < 0.05) lower increase in QTcB value from baseline to the final treatment day (visit 2) (but not significant for QTcF; p = 0.18). Similarly, the four patients > 65 years had a significantly greater increase in QTcB value than others ≤ 65 years but only in univariate analysis with QTcB and concerned a very small number of participants (n = 4). No other factors were found to have a statistically significant association with change in QTcB/F. In the strict QTcB/F population, some trends in mean QTcB/F change were observed for factors such as ‘APQ administered at least 3 h from any meal’ and ‘alcohol consumption’, without being significant.

**Table 5:** Factors associated with the change in QTcF and QTcB from baseline to the final treatment day or strict final treatment day

	QTcF population (N = 143)				Strict QTcF population (N = 60)		QTcB population (N = 143)				Strict QTcB population (N = 60)	
	n	Change in QTcF value from baseline to the final treatment day (ms): mean [CI95%]	ANOVA: p-value and R-squared	Multivariate analysis (ANOVA, backward selection process)	n	Change in QTcF value from baseline to the strict final treatment day (ms): mean [CI95%]	n	Change in QTcF value from baseline to the final treatment day (ms): mean [CI95%]	ANOVA: p-value and R-squared	Multivariate analysis (ANOVA, backward selection process)	n	Change in QTcF value from baseline to the strict final treatment day (ms): mean [CI95%]
Total	143	18.9 [14.8; 23.0]			60	17.5 [12.6; 22.4]	143	3.3 [− 0.8; 7.4]			60	2.6 [− 3.3; 8.5]
Sex												
Male	98	18.7 [14.1; 23.4]	p = 0.90		42	17.4 [10.9; 23.8]	98	3.9 [− 1.0; 8.8]	p = 0.67		42	2.9 [− 4.4; 10.3]
Female	45	19.3 [10.9; 27.6]	R^2^ = 0.0%		18	17.7 [10.4; 25.0]	45	2.0 [− 6.0; 10.0]	R^2^ = 0.1%		18	1.7 [− 9.0; 12.5]
Age category												
< 18 years old	6	26.5 [1.0; 52.0]	p = 0.44		3	14.0 [_34.0; 62.0]	6	− 0.5 [− 19.6; 18.6]	p = 0.71		3	− 13.0 [− 35.0; 9.1]
≥ 18 years old	137	18.6 [14.4; 22.8]	R^2^ = 0.4%		57	17.6 [12.6; 22.7]	137	3.5 [− 0.8; 7.7]	R^2^ = 0.1%		57	3.4 [− 2.7; 9.5]
≤ 65 years old	139	18.4 [14.3; 22.6]	p = 0.19	Not retained	58	17.0 [12.0; 22.0]	139	2.5 [− 1.6; 6.7]	p = 0.03	Not retained	58	1.6 [− 4.3; 7.4]
> 65 years old	4	35.0 [20.9; 49.1]	R^2^ = 1.2%		2	31.0 [− 57.9; 119.9]	4	30.5 [4.4; 56.6]	R^2^ = 3.4%		2	32.0 [− 120.5; 184.5]
Ethnicity												
African	118	18.3 [13.7; 23.0]	p = 0.54		51	17.2 [11.6; 22.7]	118	2.3 [− 2.5; 7.0]	p = 0.29		51	1.6 [− 5.1; 8.3]
Others	25	21.6 [13.1; 30.2]	R^2^ = 0.3%		9	19.2 [7.9; 30.5]	25	8.1 [0.2; 16.0]	R^2^ = 0.8%		9	8.1 [− 4.2; 20.4]
Smoking status												
Never smoked	101	17.1 [12.3; 21.9]	p = 0.18	Not retained	40	15.0 [10.3; 19.7]	101	0.2 [− 4.7; 5.1]	p = 0.02	p = 0.0204	40	− 0.0 [− 6.2; 6.2]
Previous, current smoker or missing information	42	23.2 [15.3; 31.1]	R^2^ = 1.3%		17	22.4 [10.6; 34.2]	42	10.8 [3.4; 18.2]	R^2^ = 3.8%	R^2^ = 3.8%	20	7.8 [− 5.5; 20.9]
Alcohol consumption												
Never/on special occasions	115	19.3 [14.6; 23.9]	p = 0.74		45	19.3 [14.1; 24.6]	115	3.2 [− 1.4; 7.8]	p = 0.92		45	3.7 [− 2.7; 10.0]
Ex- or current consumer or missing information	28	17.5 [8.3; 26.7]	R^2^ = 0.1%		15	11.9 [− 0.7; 24.5]	28	3.8 [− 6.3; 13.8]	R^2^ = 0.0%		15	− 0.8 [− 16.1; 14.5]
APQ administered at least 3 h from any meal												
Yes	95	19.1 [14.2; 24.1]	p = 0.87		41	20.8 [14.3; 27.2]	95	1.9 [− 3.3; 7.1]	p = 0.35		41	5.3 [− 2.3; 13.0]
No or missing information	48	18.4 [10.9; 26.0]	R^2^ = 0.0%		19	10.4 [3.9; 16.8]	48	6.0 [− 0.9; 13.0]	R^2^ = 0.6%		19	− 3.4 [− 12.5; 5.6]
Patient having taken other treatments known to prolong QTc												
Yes	33	20.6 [12.0; 29.3]	p = 0.65		13	17.7 [8.0; 27.3]	33	7.3 [− 0.8; 15.4]	p = 0.30		13	8.7 [− 4.4; 21.8]
No	110	18.4 [13.7; 23.1]	R^2^ = 0.1%		47	17.4 [11.6; 23.2]	110	2.1 [− 2.7; 6.9]	R^2^ = 0.8%		47	0.9 [− 5.9; 7.6]
Liver abnormalities at baseline												
Yes	25	15.9 [5.9; 25.9]	p = 0.51		12	11.8 [− 3.0; 26.6]	25	1.9 [− 6.9; 10.6]	p = 0.76		12	2.3 [− 13.1; 17.8]
No or missing information	118	19.5 [15.0; 24.1]	R^2^ = 0.3%		48	18.9 [13.7; 24.0]	118	3.6 [− 1.1; 8.3]	R^2^ = 0.1%		48	2.6 [− 4.0; 9.2]
Renal abnormalities at baseline												
Yes	3	27.7 [2.8; 52.5]			2	32.5 [27.0; 38.0]	3	23.7 [− 27.3; 74.6]			2	34.0 [− 93.1; 161.1]
No or missing information	140	18.7 [14.6; 22.9]			58	16.9 [11.9; 22.0]	140	2.9 [− 1.3; 7.0]			58	1.5 [− 4.4; 7.4]

QTcF/B: corrected QT interval with Fridericia’s or Bazett’s formulae, QTcF/B population: patients with corrected QT interval calculated using Fridericia’s formulae/Bazett’s formulae, ms: millisecond, CI95%: 95% confidence interval, R^2^: R-squared, APQ: artenimol-piperaquine; Strict QTcF/B population: patients with corrected a QT interval calculated at the strict final treatment day

### Blood chemistry markers

Changes in ALAT, ASAT and creatinine from baseline to final treatment day were not significant. Factors associated with these changes are presented in Additional file 5. Univariate and multivariate analyses showed that the increase in ALAT value between baseline and visit 2 was significantly (p < 0.05) smaller among people who never smoked.

## Discussion

Safety of APQ was studied in patients with diagnosis of uncomplicated acute *P. falciparum* malaria using a post-registration longitudinal registry in 15 HCP centres of 6 European countries. One in four participants reported a total of 129 AEs. Of the AEs, 46 (11 serious) were suspected by the clinician providing care to be related to APQ, the most common being gastrointestinal disorders. There were 28 AE of special interest (cardiotoxicity, neurotoxicity and phototoxicity), predominantly cardiological (n = 21) (7.1%), therefore it was not possible to analyse the factors associated with the development of neurotoxicity and phototoxicity events.

Focusing on the cardiovascular safety outcome, on QTcF corrections, QT prolongation was observed in 5/143 participants serially assessed (11.9%), with no clinical symptoms. An increase of > 60 ms occurred in 9 participants (6.3%). Almost all of the QTc prolongations were less than 500 ms and judged as non-serious AE. In the two patients with QT prolongation over 500 ms following the first dose of APQ, the maximum value was 531 ms (QTcB). An association was observed between older (age > 65 years) individuals, and increase in QTcB, but with only four patients > 65 years it is difficult to draw conclusions without additional work. Furthermore, these differences were not observed with QTcF adjustments. One case of spontaneously reverting ventricular tachycardia, developed in a black male with a known cardiomyopathy, 5 days after his last incomplete course of APQ, and was felt by his HCP, to be unrelated to the APQ treatment.

These findings are comparable to those observed during Eurartesim^®^ development in the DM040011 study (n = 1038 subjects; prolonged QTcB and QTcF intervals were observed in 8.6% and 4.7% of patients, respectively) and in the DM040010 study (n = 756 subjects; prolonged QTcB intervals were observed in 9.1% of patients, and QTcB increase > 60 ms in 2.7% patients). In these studies, only 7 patients showed QTcF > 500 ms (0.4%) [13, 14].

In a large prospective study [19] in four African countries evaluating the clinical safety of APQ among 10,925 uncomplicated malaria cases, 797 adverse events were reported (5%), mainly infections (3.24%) and gastrointestinal disorders (1.37%). Within this study, a nested cohort of 1002 patients (161 adults and 841 children) who had completed three doses of APQ and who had complete cardiac monitoring with repeated ECGs, 89 patients (8.9%) had an increase in the QTcF compared to their baseline measurements. On day 3 pre- and post-intake, 70 and 89 patients, respectively, had a QTcF increase of ≥ 60 ms compared to their baseline, but returned to nearly baseline values on day 7. No patient had QTcF > 500 ms prior to day 3. Three patients had QTcF > 500 ms (509 ms, 501 ms, 538 ms) 3–4 h after intake of the last dose. All the QTcF values in the three patients had returned to < 500 ms on day 7 (470 ms, 442 ms, 411 ms).

In another analysis of Ghanaian data of this effectiveness and safety African platform (INDEPTH-Network), safety analysis of APQ among 4563 patients (16.0% > 18 years old) did not identify any serious safety concerns [20]. Incidence of AEs was 7.6% (11.8% for participants > 18 years old), mostly infections (4.6%) and gastrointestinal disorders (1.0%). Only 3/477 (0.6%) patients had QTcF above the 500 ms cutoff value and 38/477 (8.0%) an QTcF increase > 60 ms, which were not clinically significant. Another post-licensure study of INDEPTH -Network conducted among 1147 patients (18.3% ≥ 18 years) from four African countries found QTcF values > 500 ms on day 3 in 37 (3.2%) of participants, none of which was clinically relevant [21]. Further, in an open label trial conducted in Malawi and Mozambique on HIV-infected patients with uncomplicated malaria who were on efavirenz or nevirapine-based treatment, increase QT interval > 60 ms from baseline occurred in 31.2% (48/154) and 13.3% (8/60) of the patients, respectively. These were not clinically significant and resolved spontaneously over time [22]. In a randomized, multicentre, clinical trial conduct in three countries in Africa (7% ≥ 15 years old), QTcF increase of more than 60 ms occurred in 11% of participants treated with APQ and 6/797 (0.8%) had a post-dose QTcF value longer than 500 ms [23, 24].

Results in patients from non-endemic European countries are, therefore, consistent with these studies from Africa or Asia, which describe infrequent episodes of reversible QTc prolongation with no clinical impact. Among other observations, changes in ALAT and ASAT were of limited interest given the small change in values between groups. No factors were significantly associated with the changes in creatinine values.

Overall, few serious adverse event and no new safety signals were detected. At the multivariate analysis, AEs were significantly more often encountered among women and patients of non-African origin. This is the first time this association was recognized. This is challenging to interpret particularly where the outcome is AEs rather than adverse drug reactions. This could be related to disease manifestations in non-immune populations. Further research in these populations would be required to confirm and better understand these differences.

The study showed a cure rate of 99.2%, in line with the expected APQ efficacy [13, 14].

The strength of the study is to be a registry-based, series of patients with imported malaria exposed to APQ in six European countries with a systematic collection of all AEs in a context where concerns arise about the tolerance of anti-malarial treatments in Europe. It provides a better understanding of the use of APQ in people with co-morbidities. The study has also several limitations: it is not a comparative study; the Registry contained a small number of patients; hereby its statistical power is limited; not all patients had an ECG performed on the day of the last APQ intake. The number of lost to follow-up is significant and could have underestimated the frequency of AEs. However, the majority of loss to follow up occurred after the end of treatment and contributed to the analysis. Missing ECGs could produce a bias in the interpretation of ECG findings. Overall, the ultimate sample size achieved was less than the planned, and it is therefore possible that rare side effects may not have been detected due to the final small number of patients. The potassium value was not collected. Given these limitations, some differences are to be interpreted with caution.

## Conclusion

This is the first study addressing the safety profile of APQ treatment in a consistent number of patients with uncomplicated malaria imported to European countries in a context of routine medical prescribing of APQ. The description of the range of AE’s and the analysis of the QTc interval and other safety data, led to the conclusion that there was no new safety signal or notable changes in their frequency as compared to previously identified signals in endemic populations. Although transient QTc prolongations up to a maximum of 531 ms was reported in one subject, no clinical consequence was observed. The efficacy rate at over 99%, was also as expected. APQ was found to be a well-tolerated artemisinin-based combination with efficacy and safety at least equivalent to the other artemisinin-based combinations available in the EU. It is also characterized by a simple administration modality, once a day for 3 days, which favors compliance to treatment and is a valuable option for use in the first-line treatment of uncomplicated *P. falciparum* imported malaria.

## Supplementary Information

**Additional file 1. **Illnesses and symptoms at Visit 1 by System Organ Class and Preferred Term—safety registry of malaria patients treated with artenimol–piperaquine.

**Additional file 2. **Baseline biological results—safety registry of malaria patients treated with artenimol–piperaquine.

**Additional file 3. **Overview of adverse events (AE) and serious adverse event (SAE) other than adverse event of special interest (AESI).

**Additional file 4. **Factors associated with the occurrence of adverse event (AE) including adverse event of special interest (AESI).

**Additional file 5. **Factors associated with the change in Alanine amino-transferase (ALAT), Aspartate amino-transferase (ASAT) and creatinin from baseline to the final treatment day.

## Data Availability

All relevant data for our analyses are fully described in the paper and can be made available on request.
