# Supplementary material for: Longitudinal study based on a safety registry for malaria patients treated with artenimol–piperaquine in six European countries
Source: Malar J. 2021 May 8;20:214. doi: 10.1186/s12936-021-03750-x (PMC8105939; doi:10.1186/s12936-021-03750-x)
Supplement: Supplementary file 1 — Additional file 1. Illnesses and symptoms at Visit 1 by System Organ Class and Preferred Term—safety registry of malaria patients treated with artenimol–piperaquine. [file 12936_2021_3750_MOESM1_ESM.docx]

Additional file 1. Illnesses and symptoms at Visit 1 by System Organ Class and Preferred Term - safety registry of malaria patients treated with artenimol-piperaquine

| System Organ Class     Preferred Term | Patients having  at least one illness  or symptom reported | | |
| --- | --- | --- | --- |
|  | Patients * N=124 | | Illness/Symptom N=247 |
| Blood and lymphatic system disorders | 3 | (2.4%) | 3 |
| Cardiac disorders | 8 | (6.5%) | 9 |
| Atrial fibrillation | 2 | (1.6%) | 2 |
| Ischaemic cardiomyopathy | 2 | (1.6%) | 2 |
| Arrhythmia | 1 | (0.8%) | 1 |
| Cardiac hypertrophy | 1 | (0.8%) | 1 |
| Left ventricular hypertrophy | 1 | (0.8%) | 1 |
| Pericardial haemorrhage | 1 | (0.8%) | 1 |
| Right atrial hypertrophy | 1 | (0.8%) | 1 |
| Congenital, familial and genetic disorders | 6 | (4.8%) | 6 |
| Hypothyroidism | 5 | (4.0%) | 5 |
| Eye disorders | 3 | (2.4%) | 3 |
| Gastrointestinal disorders | 13 | (10.5%) | 14 |
| General disorders and administration site conditions | 9 | (7.3%) | 9 |
| Hepatobiliary disorders | 3 | (2.4%) | 3 |
| HIV infection | 18 | (14.5%) | 18 |
| Others infections | 36 | (29.0%) | 51 |
| Metabolism and nutrition disorders | 21 | (16.9%) | 22 |
| Diabetes mellitus | 13 | (10.5%) | 13 |
| Dyslipidaemia | 2 | (1.6%) | 2 |
| Hypercholesterolaemia | 2 | (1.6%) | 2 |
| Type 2 diabetes mellitus | 2 | (1.6%) | 2 |
| Hypokalaemia | 1 | (0.8%) | 1 |
| Overweight | 1 | (0.8%) | 1 |
| Pica | 1 | (0.8%) | 1 |
| Musculoskeletal and connective tissue disorders | 4 | (3.2%) | 4 |
| Osteoarthritis | 2 | (1.6%) | 2 |
| Arthralgia | 1 | (0.8%) | 1 |
| Back pain | 1 | (0.8%) | 1 |
| Neoplasms benign, malignant and unspecified (incl cysts and polyps) | 5 | (4.0%) | 6 |
| Uterine leiomyoma | 2 | (1.6%) | 2 |
| Acoustic neuroma | 1 | (0.8%) | 1 |
| Colon cancer | 1 | (0.8%) | 1 |
| Hepatocellular carcinoma | 1 | (0.8%) | 1 |
| Urinary bladder polyp | 1 | (0.8%) | 1 |
| Nervous system disorders | 9 | (7.3%) | 9 |
| Headache | 3 | (2.4%) | 3 |
| Epilepsy | 2 | (1.6%) | 2 |
| Hypoaesthesia | 1 | (0.8%) | 1 |
| Parkinson's disease | 1 | (0.8%) | 1 |
| Sciatica | 1 | (0.8%) | 1 |
| VIIth nerve paralysis | 1 | (0.8%) | 1 |
| Pregnancy, puerperium and perinatal conditions | 1 | (0.8%) | 2 |
| Abortion spontaneous | 1 | (0.8%) | 2 |
| Psychiatric disorders | 3 | (2.4%) | 3 |
| Depression | 1 | (0.8%) | 1 |
| Major depression | 1 | (0.8%) | 1 |
| Psychomotor retardation | 1 | (0.8%) | 1 |
| Renal and urinary disorders | 3 | (2.4%) | 3 |
| Nephrotic syndrome | 1 | (0.8%) | 1 |
| Renal failure | 1 | (0.8%) | 1 |
| Urinary incontinence | 1 | (0.8%) | 1 |
| Reproductive system and breast disorders | 1 | (0.8%) | 1 |
| Benign prostatic hyperplasia | 1 | (0.8%) | 1 |
| Respiratory, thoracic and mediastinal disorders | 9 | (7.3%) | 9 |
| Asthma | 8 | (6.5%) | 8 |
| Asthma exercise induced | 1 | (0.8%) | 1 |
| Skin and subcutaneous tissue disorders | 2 | (1.6%) | 2 |
| Hyperhidrosis | 1 | (0.8%) | 1 |
| Rash | 1 | (0.8%) | 1 |
| Surgical and medical procedures | 6 | (4.8%) | 11 |
| Vascular disorders | 48 | (38.7%) | 52 |
| Hypertension | 47 | (37.9%) | 47 |
| Aortic stenosis | 1 | (0.8%) | 1 |
| Haemorrhoids | 1 | (0.8%) | 1 |
| Hypotension | 1 | (0.8%) | 1 |
| Ischaemic stroke | 1 | (0.8%) | 1 |
| Shock haemorrhagic | 1 | (0.8%) | 1 |

*If one patient presented an illness/symptom several times (same preferred term), s/he is counted once for that term.
