# Supplementary material for: Longitudinal study based on a safety registry for malaria patients treated with artenimol–piperaquine in six European countries
Source: Malar J. 2021 May 8;20:214. doi: 10.1186/s12936-021-03750-x (PMC8105939; doi:10.1186/s12936-021-03750-x)
Supplement: Supplementary file 2 — Additional file 2. Baseline biological results—safety registry of malaria patients treated with artenimol–piperaquine. [file 12936_2021_3750_MOESM2_ESM.docx]

Additional file 2. Baseline biological results - safety registry of malaria patients treated with artenimol-piperaquine

|  | Total | |
| --- | --- | --- |
|  | N=294 | |
| Number of patients for whom at least one baseline haematological result is available | 285 | (96.9%) |
| Hemoglobin (g/dL) |  |  |
| N | 285 | |
| Mean (SD) | 12.77 (2.10) | |
| Below lower normal range | 109 | (38.2%) |
| Hematocrit (%) |  |  |
| N | 279 | |
| Mean (SD) | 37.85 (6.02) | |
| Below lower normal range | 111 | (39.8%) |
| Leukocytes (G/L) |  |  |
| N | 278 | |
| Mean (SD) | 5.288 (1.852) | |
| Below lower normal range | 69 | (24.8%) |
| Neutrophil granulocytes (%) |  |  |
| Below lower normal range | 26/259 | (10.0%) |
| Lymphocytes (%) |  |  |
| Below lower normal range | 68/255 | (26.7%) |
| Eosinophil granulocytes (%) |  |  |
| Above upper normal range | 9/255 | (3.5%) |
| Platelet (G/L) |  |  |
| Median | 89.0 | |
| Q1;Q3 | 59.0 ; 135.0 | |
| Below lower normal range | 213 | (75.3%) |
| C-Reactive Protein (mg/L) |  |  |
| Median | 94.0 | |
| Q1;Q3 | 33.0 ; 153.0 | |
| Above upper normal range | 227/241 | (94.2%) |
| Glucose (mg/dL) |  |  |
| N | 197 | |
| Mean (SD) | 117.81 (43.48) | |
| Above upper normal range | 104 | (52.8%) |
| Creatinine (mg/L) |  |  |
| N | 278 | |
| Mean (SD) | 10.44 (7.71) | |
| Above upper normal range | 74 | (26.6%) |
| Total Bilirubin (mg/dL) |  |  |
| N | 239 | |
| Mean (SD) | 1.513 (0.931) | |
| Above upper normal range | 125 | (52.3%) |
| Aspartate Amino-transferase (mIU/mL) |  |  |
| N | 247 | |
| Mean (SD) | 43.4 (23.8) | |
| Above upper normal range | 97 | (39.3%) |
| Alanine Amino-transferase (mIU/mL) |  |  |
| N | 267 | |
| Mean (SD) | 40.5 (25.4) | |
| Above upper normal range | 75 | (28.1%) |
| Alkaline Phosphatase (mIU/mL) |  |  |
| N | 168 | |
| Mean (SD) | 77.4 (28.1) | |
| Above upper normal range | 15 | (8.9%) |
| Gamma Glutamyl Transferase (mIU/mL) |  |  |
| N | 179 | |
| Mean (SD) | 69.7 (70.3) | |
| Above upper normal range | 81 | (45.3%) |

Baseline assessments: assessments reported at visit 1 with date of blood test <= date of the first administration of APQ
or assessments reported at Visit 2 with date of blood test < date of the first APQ administration.
