# Supplementary material for: Longitudinal study based on a safety registry for malaria patients treated with artenimol–piperaquine in six European countries
Source: Malar J. 2021 May 8;20:214. doi: 10.1186/s12936-021-03750-x (PMC8105939; doi:10.1186/s12936-021-03750-x)
Supplement: Supplementary file 3 — Additional file 3. Overview of adverse events (AE) and serious adverse event (SAE) other than adverse event of special interest (AESI). [file 12936_2021_3750_MOESM3_ESM.docx]

Additional file 3: Overview of adverse events (AE) and serious adverse event (SAE) other than adverse event of special interest (AESI)

| Type of AE other than AESI | AE | SAE | AE suspected related to AP | SAEs suspected related to APQ |
| --- | --- | --- | --- | --- |
|  |  |  |  |  |
| Total number of AEs/SAEs other than AESIs  Intensity  Mild  Moderate  Severe  Outcome  Study drug dosage adjusted  Study drug permanently discontinued due to this AE  Hospitalization / prolongation of hospitalization  Relationship with artenimol-piperaquine  Suspected  Unsuspected | 129  91 (70.5%)  29 (22.5%)  9 (7.0%)  1 (0.8%)  5 (3.9%)  25 (19.4%)  46 (35.7%)  83 (64.3%) | 27  7 (25.9%)  12 (44.4%)  8 (29.6%)  0 (0.0%)  4 (14.8%)  25 (92.6%)  11 (40.7%)  16 (59.3%) | 46 | 11 |
| System organ class or preferred term (MedDRA)  Blood and lymphatic system disorders (Aenemia, Haemolysis, Eosinophilia, Leukocytosis, Lymphadenopathy, Thromocytopenia)  Cardiac disorders (Athletic heart syndrome, oedema peripheral, right atrial hypertrophy)  Ear disorders (Ear pain, hypoacusis)  Eye disorders (Blepharitis, diplopia)  Gastrointestinal disorders (vomiting, diarrhoea, abdominal pain, nausea, oropharyngeal pain, gastrointestinal disorder or pain)  General disorders (Asthenia, pyrexia, malaise, fatigue, hyperthermia, influenza like illness, night sweats)  Hepatobiliary disorders (Cholelithiasis, hepatitis, hepatitis acute, hepatocellular injury)  Infections* (related: encephalitis, malaria^§^)  Injury (cinchonism)  Investigations  Metabolism (hyperkalaemia)  Musculoskeletal and connective tissue disorders  Neoplasm (uterine leiomyoma)  Nervous system disorder (headache, balance disorder, confusion, insomnia, meningism)  Acute renal failure  Gynecology (polymenorrhoea)  Respiratory disorders (cough, dyspnoea, rhinorrhoea)  Skin disorders (pruritus, dry skin)  Hypotension | 11 (8.5%)  3 (2.3%)  2 (1.6%)  2 (1.6%)  29 (22.5%)  16 (12.4%)  4 (3.1%)  14 (10.9%)  1 (0.8%)  2 (1.6%)  1 (0.8%)  4 (3.1%)  1 (0.8%)  21 (16.3%)  1 (0.8%)  1 (0.8%)  12 (9.3%)  3 (2.3%)  1 (0.8%) | 5 (18.5%)  1 (14.2%)  5 (8.8%)  3 (11.1%)  2 (7.4%)  6 (10.5%)  4 (7.0%)  1 (3.7%) | 4 (8.7%)  20 (43.4%)  2 (4.3%)  2 (4.3%)  3 (6.5%)  4 (8.7%)  7 (15.2%)  2 (4.3%)  2 (4.3%) | 3 (27.3%)  3 (27.3%)  1 (9.1%)  2 (18.2%)  2 (18.2%) |

An event is considered as suspected related to APQ if the relationship with APQ is 'related', 'suspected', 'unassessable' or 'missing' as reported by the investigator in the 'Adverse event' section of the CRF.

AE: Adverse event, SAE: Serious adverse event, AESI: Adverse event of special interest, APQ: artenimol-piperaquine, MedDRA: AEs are coded using MedDRA dictionary version 16.0.

*Bronchitis, pneumonia, atypical pneumonia, encephalitis brain stem, malaria, nasopharyngitis, oral herpes, plasmodium falciparum infection, pneumonia bacterial, pyelonephritis, respiratory tract infection

^§^Although to say that malaria is an adverse reaction related to APQ is contradictory, the declaration corresponds to that of the claims of the investigators
