# Supplementary material for: Longitudinal study based on a safety registry for malaria patients treated with artenimol–piperaquine in six European countries
Source: Malar J. 2021 May 8;20:214. doi: 10.1186/s12936-021-03750-x (PMC8105939; doi:10.1186/s12936-021-03750-x)
Supplement: Supplementary file 4 — Additional file 4. Factors associated with the occurrence of adverse event (AE) including adverse event of special interest (AESI). [file 12936_2021_3750_MOESM4_ESM.docx]

Additional file 4: Factors associated with the occurrence of adverse event (AE) including adverse event of special interest (AESI)

|  | Adverse Events (N=294) | | | | Adverse events suspected related to APQ (N=294) | | |
| --- | --- | --- | --- | --- | --- | --- | --- |
|  |  | At least one AE (%) | Mixed logistic regression with centre as random factor | |  | At least one AE suspected related to APQ (%) | Pearson's Chi-squared test: p-value |
|  |  |  | Univariate: P-value | Multivariate with Backward selection process. Adjusted Odds-Ratio [95% CI] P-value |  |  |  |
|  | n |  |  | N=293 | n |  |  |
| Total | | | | | | | |
|  | 294 | 30.6% |  |  | 294 | 16,2% |  |
| Gender | | | | | | | |
| Male | 205 | 26.3% | p=0.06 | ref. | 205 | 13,7% | p=0.15 |
| Female | 89 | 40.4% |  | aOR=1.78 [1.00;3.16], p=0.048 | 89 | 20,2% |  |
| Age catgegory^1^ | | | | | | | |
| <= 12 years old | 5 | 0,0% |  |  | 5 | 0,0% |  |
| 13-17 years old | 5 | 0,0% |  |  | 5 | 0,0% |  |
| >= 18 years old | 284 | 30.6% |  |  | 284 | 16,2% |  |
| Ethnicity | | | | | | | |
| African | 248 | 26.2% | p=0.004 | ref. | 248 | 13,7% | p=0.03 |
| Others | 45 | 55.6% |  | aOR=2.91 [1.44;5.87], p=0.003 | 45 | 26,7% |  |
| Smoking status | | | | | | | |
| Never smoked | 205 | 27.3% | p=0.16 | Not retained | 205 | 13,7% | p=0.15 |
| Previous, current smoker or missing information | 89 | 38.2% |  |  | 89 | 20,2% |  |
| Alcohol consumption | | | | | | | |
| Never/On special occasions (less than once in a week) | 237 | 31.2% | p=0.43 |  | 237 | 15,6% | p=0.97 |
| Ex- or current consumer or missing information | 57 | 28.1% |  |  | 57 | 15,8% |  |
| APQ administered at least 3 hours from any meal | | | | | | | |
| Yes | 182 | 26.9% | p=0.44 |  | 182 | 13,7% | p=0.25 |
| No or missing information | 112 | 36.6% |  |  | 112 | 18,8% |  |
| Patient having taken treatments known to prolong QT interval | | | | | | | |
| Yes | 81 | 33.3% | p=0.54 |  | 81 | 22,2% | p=0.06 |
| No | 213 | 29.6% |  |  | 213 | 13,1% |  |
| Liver abnormalities at baseline* | | | | | | | |
| Yes | 54 | 25.9% | p=0.28 |  | 54 | 11,1% | p=0.31 |
| No or missing information | 240 | 31.7% |  |  | 240 | 16,7% |  |
| Renal abnormalities at baseline** | | | | | | | |
| Yes | 14 | 35.7% |  |  | 14 | 35,7% | p=0.03 |
| No or missing information | 280 | 30.4% |  |  | 280 | 14,6% |  |

CI95%: 95% confidence interval, APQ: artenimol-piperaquine, aOR: adjusted Odds Ratio, AE: adverse events including AE of special interest and serious AE, ^1^ No test has been performed due to the small number of patients; Liver abnormalities at baseline: ALT=>1.5*UNL or AST=>1.5*UNL, with UNL=Upper Normal Limit.; **Renal abnormalities at baseline: Creatinin=>1.5*UNL, with UNL=Upper Normal Limit. No test has been performed due to the small number of patients with a renal abnormality at baseline
