# Supplementary material for: Longitudinal study based on a safety registry for malaria patients treated with artenimol–piperaquine in six European countries
Source: Malar J. 2021 May 8;20:214. doi: 10.1186/s12936-021-03750-x (PMC8105939; doi:10.1186/s12936-021-03750-x)
Supplement: Supplementary file 5 — Additional file 5. Factors associated with the change in Alanine amino-transferase (ALAT), Aspartate amino-transferase (ASAT) and creatinin from baseline to the final treatment day. [file 12936_2021_3750_MOESM5_ESM.docx]

Additional file 5. Factors associated with the change in Alanine amino-transferase (ALAT), Aspartate amino-transferase (ASAT) and creatinin from baseline to the final treatment day

|  | ALAT (N=294) | | | | ASAT (N=294) | | | | creatinine (N=294) | | | |
| --- | --- | --- | --- | --- | --- | --- | --- | --- | --- | --- | --- | --- |
|  |  | Change in ALAT from baseline to the Final treatment day (mIU/mL): median [IQR] | Wilcoxon's test: p-value | Multivariate analysis (ANOVA, backward selection process) |  | Change in ASAT from baseline to the Final treatment day (mIU/mL): median [IQR] | Wilcoxon's test: p-value | Multivariate analysis (ANOVA, backward selection process) |  | Change in creatinine from baseline to the Final treatment day (mg/L): median [IQR] | Wilcoxon's test: p-value | Multivariate analysis (ANOVA, backward selection process) |
|  | n |  |  | N=196 | n |  |  | N=176 | n |  |  |  |
| Total | | | | | | | | | | | | |
|  | 294 | 0.0 [-5.0;8.0] |  |  | 294 | -1.0 [-7.5;6.0] |  |  | 294 | -0.7 [-1.8;0.2] |  |  |
| Gender | | | | | | | | | | | | |
| Male | 205 | 0.0 [-5.0;11.0] | p=0.65 |  | 205 | - 1.0 [-9.0;5.0] | p=0.63 |  | 205 | - 0.8 [-1.9;0.4] | p=0.88 |  |
| Female | 89 | - 0.5 [-6.0;6.0] |  |  | 89 | 0.0 [-7.0;9.0] |  |  | 89 | - 0.5 [-1.6;0.0] |  |  |
| Age catgegory^1^ | | | | | | | | | | | | |
| <= 12 years old | 5 | 0.0 [0.0;0.0] |  |  | 5 | 0.0 [0.0;0.0] |  |  | 5 | 0.2 [0.2;0.2] |  |  |
| 13-17 years old | 5 | - 14.0 [-35.0;71.0] |  |  | 5 | - 14.5 [-22.5;100.0] |  |  | 5 | - 0.4 [-0.9;0.3] |  |  |
| >= 18 years old | 284 | 0.0 [-5.0;8.0] |  |  | 284 | - 1.0 [-7.5;6.0] |  |  | 284 | - 0.7 [-1.8;0.2] |  |  |
| Ethnicity | | | | | | | | | | | | |
| African | 248 | -1.0 [-5.0;5.0] | p=0.05 | Not retained | 248 | - 1.0 [-9.0;4.0] | p=0.04 | p=0.04 | 248 | - 0.7 [-1.8;0.2] | p=0.94 |  |
| Others | 45 | 6.0 [-4.0;26.0] |  |  | 45 | 5.0 [-4.0;35.0] |  |  | 45 | - 0.8 [-1.6;0.2] |  |  |
| Smoking status | | | | | | | | | | | | |
| Never smoked | 205 | - 1.0 [-6.0;5.0] | p=0.003 | p=0.002 | 205 | - 2.0 [-9.0;3.0] | p=0.02 | p=0.05 | 205 | - 0.7 [-1.8;0.2] | p=0.40 |  |
| Previous, current smoker or missing information | 89 | 3.0 [-2.0;14.0] |  |  | 89 | 1.5 [-5.0;8.0] |  |  | 89 | - 0.5 [-1.6;0.4] |  |  |
| Alcohol consumption | | | | | | | | | | | | |
| Never/On special occasions (less than once in a week) | 237 | 0.0 [-5.0;7.0] | p=0.93 |  | 237 | - 1.0 [-8.0;5.0] | p=0.90 |  | 237 | - 0.7 [-1.9;0.2] | p=0.49 |  |
| Ex- or current consumer or missing information | 57 | - 1.0 [-6.0;10.0] |  |  | 57 | - 1.0 [-9.0;12.0] |  |  | 57 | - 0.7 [-1.3;0.5] |  |  |
| APQ administered at least 3 hours from any meal | | | | | | | | | | | | |
| Yes | 182 | 0.0 [-4.0;7.0] | p=0.60 |  | 182 | - 1.0 [-7.0;8.0] | p=0.52 |  | 182 | - 0.6 [-1.7;0.2] | p=0.60 |  |
| No or missing information | 112 | 0.0 [-7.0;10.0] |  |  | 112 | - 1.0 [-4.8;8.7] |  |  | 112 | - 0.7 [-2.0;0.2] |  |  |
| Patient having taken other treatments known to prolong QT | | | | | | | | | | | | |
| Yes | 81 | 2.0 [-4.0;17.0] | p=0.05 | p=0.04 | 81 | 0.0 [-7.0;15.0] | p=0.26 |  | 81 | - 0.5 [-2.0;0.4] | p=0.60 |  |
| No | 213 | - 1.0 [-5.0;5.0] |  |  | 213 | - 1.0 [-8.0;6.0] |  |  | 213 | - 0.7 [-1.8;0.1] |  |  |
| Liver abnormalities at baseline* | | | | | | | | | | | | |
| Yes | 54 | 1.5 [-19.0;18.0] | p=0.65 |  | 54 | - 14.0 [-27.0;10.0] | p=0.03 | p=0.01 | 54 | - 0.5 [-1.8;0.4] | p=0.71 |  |
| No or missing information | 240 | - 0.5 [-4.0;6.0] |  |  | 240 | - 1.0 [-6.0;5.0] |  |  | 240 | - 0.7 [-1.8;0.2] |  |  |
| Renal abnormalities at baseline** | | | | | | | | | | | | |
| Yes | 14 | - 3.5 [-9.0;2.5] |  |  | 14 | - 4.0 [-6.0;11.0] |  |  | 14 | - 2.8 [-4.1;1.3] |  |  |
| No or missing information | 280 | 0.0 [-5.0;9.5] |  |  | 280 | - 1.0 [-8.0;6.0] |  |  | 280 | - 0.6 [-1.7;0.2] |  |  |

^1^ No test has been performed due to the small number of patients aged < 18 years old; *Liver abnormalities at baseline: ALT=>1.5*UNL or AST=>1.5*UNL, with UNL=Upper Normal Limit.; **Renal abnormalities at baseline: Creatinine=>1.5*UNL, with UNL=Upper Normal Limit. No test has been performed due to the small number of patients with a renal abnormality at baseline; IQR: Interquartile range

No substantial changes in haemoglobin and haematocrit were observed. Neutrophil granulocyte, platelet counts, liver parameters and C-reactive protein tended to improve during the course of the study from baseline to the final treatment day. Eosinophilia rates increased during the course of the study from 9/255 (3.55%) with eosinophil granulocytes above upper normal range at baseline to 29/144 (19.3%) the 21^st^ day or beyond. No factors were significantly associated with the changes in creatinine values. Changes in ALAT, ASAT and creatinine from baseline to final treatment day were not significant in the median. Univariate and multivariate analyses showed that the increase in ALAT value between baseline and visit 2 was significantly smaller among people who never smoked and larger among patients receiving treatments known to prolong QT. In addition, people who never smoked, patients with liver abnormalities at baseline and African patients were more likely to have a smaller change in Aspartate Amino-Transferase value from baseline to visit 2.
